# Thyrotoxic Periodic Paralysis in a Samoan Male With Metabolic Acidosis: A Case Report and Review of the Literature

**DOI:** 10.7759/cureus.65309

**Published:** 2024-07-24

**Authors:** Lauren A Nguyen, Marguerite Cazin, J. Douglas Miles

**Affiliations:** 1 Department of Neurology, John A. Burns School of Medicine, University of Hawaii, Honolulu, USA

**Keywords:** pacific-islander, paralysis, thyrotoxic periodic paralysis, hypokalemia, hyperthyroidism

## Abstract

Thyrotoxic periodic paralysis (TPP) is a rare disorder characterized by muscle paralysis, thyrotoxicosis, and hypokalemia. It commonly manifests as paralysis of both proximal and distal upper and lower limbs, and if left untreated, may progress to respiratory failure or cardiac arrhythmias. It is most common in Asian males and is frequently precipitated by strenuous exercise, high carbohydrate diet, stress, corticosteroid therapy, or alcohol. Early diagnosis of TPP is crucial as the condition may be reversible with oral or IV potassium replacement therapy, and management of the underlying hyperthyroidism. We describe a Samoan man in his 30s who presented with acute onset lower extremity paralysis. Laboratory investigations revealed low serum potassium of 2.2 mEq/L (reference range 3.5-5.0 mEq/L) and thyrotoxicosis with a low (thyroid stimulating hormone (TSH) of <0.07 uIU/mL (reference range 0.27-4.20 uIU/mL) and an elevated free T4 of 5.4 ng/dL (reference range 0.9-2.1 ng/dL). He was treated with both oral and IV potassium chloride as well as propranolol and regained full strength in his extremities. While rare, TPP is a reversible complication of thyrotoxicosis and a high index of suspicion in clinical practice is essential to prevent adverse outcomes.

## Introduction

Thyrotoxic periodic paralysis (TPP) is a rare, life-threatening complication of hyperthyroidism, characterized by acute and reversible episodes of muscle weakness and hypokalemia in hyperthyroid patients. Occurring in 0.1-0.2% of the hyperthyroid population of North America, TPP is more frequent in males and in Asian populations, manifesting in about 2% of the hyperthyroid Asian population [1]. Within the Asian population, it has been primarily characterized in Chinese, Japanese, Vietnamese, Filipino, and Korean ethnicities [2,3].

TPP is rarely diagnosed at first attack due to its mild, nonspecific symptoms [2]. Factors such as a high carbohydrate diet, alcohol, strenuous exercise, emotional stress, or steroid use can precipitate episodes of TPP [3-5]. We present a case of a Samoan man in his 30s residing in Hawaii who presented to the emergency department with acute bilateral lower extremity weakness and hypokalemia and was subsequently diagnosed with TPP. As TPP is primarily seen in Asian men, this presentation in a Polynesian man is rare in the United States. Additionally, the patient presented with metabolic acidosis, which is another rare complication of TPP. In this case report, we also review the common presenting signs and symptoms, diagnosis, and treatment of TPP. 

## Case presentation

A male of Samoan ethnicity in his 30s, residing in Hawaii, with no significant contributory past medical history presented to our hospital with weakness that began in his proximal to distal lower extremities two days prior. The weakness became progressively worse with occasional numbness, resulting in a fall with no head trauma the day prior to presentation. The weakness subsequently expanded to his arms, and he stated being unable to move all four extremities against gravity. He complained of multiple associated episodes of watery diarrhea without abdominal pain or nausea, which began the day of his ED presentation. He denied any back pain or speech changes. He denied any recent COVID-19 or flu-like symptoms and taking any weight loss supplements or thyroid/hormone medications. No triggers such as emotional stress, trauma, high carbohydrate meals, alcohol, or steroid use were noted. He reported experiencing one similar episode approximately one month and a half prior, where he developed severe bilateral lower extremity weakness. It was severe enough that he could not walk, yet his strength improved spontaneously.

On examination, his blood pressure was 188/86 mmHg with a pulse of 108. He was afebrile with no significant abdominal exam findings. His neurologic exam revealed full 5/5 strength in his upper extremities but 3/5 strength in his lower extremities. His sensations were intact to light touch. Patellar deep tendon reflexes were decreased. 

At this point, the differential diagnosis was broad but included metabolic derangement and Guillain-Barre syndrome. A non-contrast head CT revealed no intracranial hemorrhage, no acute infarct, and no ventriculomegaly. A lumbar puncture was performed; CSF studies were normal. Because Guillain-Barre syndrome was initially suspected, titers of GQ1b antibodies (IgG) and GM-1 antibodies were ordered. However, both antibodies came back negative (Table 1). 

**Table 1:** Antibody tests ordered in the emergency department GM-1 Ab (IgG): ganglioside GM1 antibody (immunoglobulin G); GQ1b Ab (IgG): ganglioside GQ3 antibody (immunoglobulin G); TSH: thyroid stimulating hormone; TPO: thyroid peroxidase; H: high

Antibody	Result	Reference Range
GM-1 Ab (IgG)	<1:800	Less than 1:800
GQ1b Ab (IgG)	<1:100	Less than 1:100
TSH receptor antibody	9.18 (H)	
Anti-TPO microsomal	189 (H)	<34 IU/mL

A complete blood count (CBC) and basic metabolic panel (BMP) were ordered. Potassium was extremely low at 2.2 mEq/L, glucose was elevated at 253 mg/dL, and CO_^2^_ was low at 20 mEq/L (Table 2). A blood-gas panel showed decreased pH (7.28), high pCO_2_ (43 mmHg), low pO_2_ (low mmHg), base excess decreased (-6 mmol/L), decreased HCO_3_^-^ (20 mmol/L), decreased O_2_ saturation at 87%, decreased potassium of <1.5 mEq/L, and increased calcium at 1.43 mg/L (Table 3). The CBC was unremarkable. 

**Table 2:** Serum labs ordered in the emergency department, including thyroid function tests and potassium levels. CO_2_: carbon dioxide; BUN: blood urea nitrogen; T4: thyroxine; TSH: thyroid stimulating hormone

Laboratory Component	Value	Reference Range
Lipase	39	18-71 U/L
Sodium	138	133-145 mmol/L
Potassium	2.2 (LL)	3.3-5.1 mEq/L
Chloride	105	95-108 mEq/L
CO_2_	20	21-30 meQ/L
Calcium	9.5	8.3-10.5 mg/L
Anion Gap	15	14-20 mEq/L
Glucose	253	70-99 mg/dL
BUN	13	6-23 mg/L
Creatinine	0.6	0.6-1.4 mg/dL
White blood cell count	4.92	3.80-10.80 x10^3 uL
Red blood cell count	5.09	4.00-6.20 x 10^6/uL
Hemoglobin	13.7	13.7-17.5 g/dL
Hematocrit	42.2	40.1-51%
Troponin T Gen 5	7	<19 ng/L
Magnesium	1.8	1.6-2.6 mg/dL
Free T4	5.4 (H)	0.9-2.1 ng/dL
TSH	<0.07 (L)	0.27-4.20 uIU/mL

**Table 3:** Blood-gas panel ordered in the emergency department. pCO_2_: partial pressure of carbon dioxide; pO_2_: partial pressure of oxygen; O_2_ sat: oxygen saturation; L: low; H: high

Laboratory Component	Value	Reference Range
pH	7.28 (L)	7.38-7.42
pCO_2_	43 (H)	38-42 mmHg
pO_2_	59 (L)	80-100 mmHg
Base excess	-6 (L)	(-2)-(+2) mmol/L
HCO_3_^-^	20 (L)	22-26 mmol/L
O_2 _Sat (Calc.)	87 (L)	OR >97%

An EKG was ordered for his hypokalemia and demonstrated sinus tachycardia at 105 bpm, consistent with the patient’s most recent EKG from two years prior. A urine drug screen was negative. A chest X-ray found no pulmonary edema or cardiomegaly.

**Figure 1:**
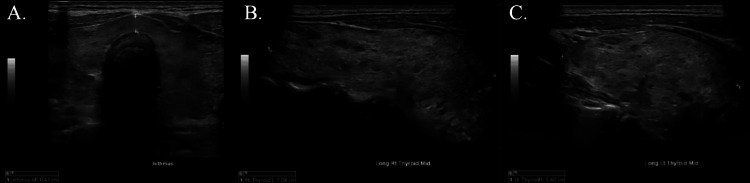
Thyroid ultrasound (A) Transverse image of the thyroid gland. (B) Longitudinal image of the left thyroid measuring 5.60 cm in length, showing enlargement with diffuse heterogeneous echotexture. (C) Longitudinal image of the right thyroid measuring 7.08 cm in length, showing enlargement with diffuse heterogeneous echotexture.

After ruling out other causes for the patient’s severe hypokalemia, thyrotoxic periodic paralysis was suspected. A thyroid panel was ordered and revealed decreased TSH at 0.07 and increased free T4 at 5.4 (Table 2). Anti-TPO and TSH receptor antibodies were ordered, both of which were elevated at 189 and 9.18, respectively (Table 1). Ultrasound of the thyroid revealed a diffusely enlarged thyroid with heterogenous echotexture, but no suspicious nodules were identified (Figure 1). Given the lack of other pertinent symptoms, the patient was diagnosed with thyrotoxic periodic paralysis. 

The patient was admitted to the hospital and was administered both oral and IV potassium chloride as well as a magnesium sulfate IV. His strength returned to normal and he reported feeling a complete resolution of his symptoms. The potassium chloride was then tapered off to avoid the risk of rebound hyperkalemia. He was prescribed propranolol as well as methimazole and was educated on the importance of avoiding high-carbohydrate meals, heavy exercise, and alcohol. He was discharged and instructed to continue regular follow-ups with both an endocrinologist and a neurologist. 

## Discussion

In this case, we present a patient who was diagnosed with TPP after presenting to the emergency department with two days of acute bilateral lower extremity weakness and hypokalemia. The patient was found to be in hypoxic metabolic acidosis, with a low pH, high pCO_^2^_, low pO_^2^_, high HCO_3_^-^, and decreased O_^2^_ saturation. To the best of our knowledge, this is the first case of metabolic acidosis associated with TPP in the United States, and the second reported case globally. The previous case associated with metabolic acidosis was of a 23-year-old Moroccan male who had reported episodes of flaccid tetraparesis for 11 months alongside chronic gastritis [6]. Similar to our patient, the man from Morocco also presented with diarrhea. However, his presentation was much different from our patients’ in that our patient had no prior history of chronic tetraparesis or chronic gastritis. In addition, Allard et al.'s patient had lactic metabolic acidosis [6]. In the case of our patient, lactate levels were not ordered at the time of diagnosis and the cause of our patient’s metabolic acidosis remains speculative. Other laboratory levels that were not ordered at the time of diagnosis, but might have been helpful in narrowing the differential diagnosis of metabolic acidosis in our patient, are total creatinine phosphokinase, uric acid, and lactate dehydrogenase levels. An acid-base imbalance is uncommon in patients with TPP, explained by the exchange of K^+^ with Na^+^ or H^+^ to maintain electroneutrality. Mild respiratory alkalosis, likely due to fear or stress-related hyperventilation, and mild respiratory acidosis, likely due to hypokalemia-induced respiratory muscle weakness, have been reported in some TPP cases [7]. 

Populations of Polynesian ethnicity experience an increased risk for TPP, with an increased prevalence of up to 37-fold in one study conducted in New Zealand [8]. The first documented case of TPP in a Polynesian patient was recorded in New Zealand by Carroll [9]. To the best of our knowledge, TPP in a Polynesian male has only been described once prior in the United States, by Brown et al. [10]. This makes the present case report the second known case of TPP in the Polynesian population in the United States. Brown et al.'s case of TPP was also reported in Hawaii [10]. Similar to our patient, their patient was also a young man who presented with diarrhea and a one-day history of bilateral leg weakness. He also complained of weakness that led to falls. Thus, in areas with large Polynesian populations such as Hawaii, TPP should be considered in clinicians’ differential diagnoses for patients presenting with these symptoms. 

The pathogenesis of TPP is thought to be associated with a hyperthyroidism-induced increase in Na^+^-K^+^-ATPase activity, resulting in an intracellular K^+^ shift. Increased β-adrenergic activity and insulin secretion induced by hyperthyroidism can additionally contribute to an increase in Na^+^-K^+^-ATPase expression. Individuals with TPP have a significantly higher Na^+^-K^+^-ATPase number and activity than both healthy subjects and hyperthyroid patients without a history of paralysis [1]. Hypokalemia develops with increased Na^+^-K^+^-ATPase activity, as well as hyperthyroidism-mediated hyperinsulinemia [11]. Genetic predispositions for TPP previously documented in the literature include mutations in the *SCN4A* (sodium voltage-gated channel alpha unit 4) gene (70% TTP cases) and in the *CACNA1s* (calcium voltage-gated channel alpha subunit 4 S) gene (10% TPP cases) [12]. 

TPP diagnosis requires evidence of hyperthyroidism and hypokalemia, as well as a possible history of recurrent episodes of proximal muscle weakness affecting the lower limbs, without family history. Hypokalemia is present in most patients, as well as hypomagnesemia and hypophosphatemia, further distinguishing TPP from familial periodic paralysis. Deep tendon reflexes are significantly diminished or absent in the majority of cases, while cognitive and sensory function remains normal [1,4,5]. These characteristic features of TPP were consistent with the patient reported in this case, who presented with two days of lower extremity weakness, severe hypokalemia at 2.2 mEq/L (reference range 3.5-5.0 mEq/L), and diminished patellar deep tendon reflexes, with subjective sensation intact. He also had a history of one similar attack one month prior, consistent with the presentation of TPP. Other symptoms consistent with hyperthyroidism at presentation include hyperglycemia, tachycardia, systolic hypertension, and multiple episodes of diarrhea, which may have further exacerbated the patient’s hypokalemia. TPP attacks can often occur during the night and after stress, alcohol intake, a carbohydrate-rich meal, high-dose glucocorticoid use, HIV antiretroviral therapy, or interferon-alpha use [3,4]. However, Chang et al. found that only 34% of patients presented with a precipitating factor [13]. In our patient, there was no evidence of alcohol intake, a carbohydrate-rich meal, glucocorticoid use, or other medication that could have precipitated this attack. However, he did experience this attack late at night, as he reported to the emergency department at approximately 4:00 am. ECG findings associated with TPP include sinus tachycardia attributable to a hyperadrenergic state, prolonged QTu interval due to hypokalemia, and a prolonged PR interval due to hyperthyroidism [3]. In the current case, the patient’s ECG demonstrated sinus tachycardia at 105 bpm, consistent with a hyperthyroid state. After obtaining a thyroid panel, the patient was found to have decreased TSH, increased free T4, and elevated anti-TPO antibodies. 

The differential diagnosis for our patient’s hyperthyroidism included multinodular goiter, Grave’s disease, or early-stage Hashimoto’s thyroiditis. While the patient’s thyroid ultrasound findings of heterogeneous echotexture and diffuse enlargement are nonspecific, they are abnormal and may or may not be suggestive of multinodular goiter, Grave's disease, or early-stage Hashimoto's thyroiditis. In addition, the presence of elevated anti-TPO and anti-TSH antibodies is also suspicious for both Grave’s disease and Hashimoto’s thyroiditis. Radioactive iodine uptake (RAIU) and thyroid scan remain the gold standard for differentiating causes of thyrotoxicosis, with sensitivity and specificity over 95% [14]. As per the joint guideline established by the American Thyroid Association and the American Association of Clinical Endocrinologists, a radioactive iodine uptake (RAIU) and thyroid scan are recommended as the primary modality for differential diagnosis in cases where the clinical manifestation of thyrotoxicosis does not definitively indicate Graves' disease [15]. 

TPP treatment involves the medical management of thyrotoxicosis and potassium administration. Preventative measures include maintenance of a euthyroid state with non-selective β-adrenergic blockers and a low-carbohydrate diet [1]. After stabilization of hypokalemia with oral and IV potassium chloride and magnesium, our patient was discharged on propranolol and methimazole.

## Conclusions

We presented the case of a young Polynesian man with TPP and hypoxic metabolic acidosis. TPP is a rare complication of thyrotoxicosis and is even more rarely complicated by metabolic acidosis, demonstrating the uniqueness of this case. Additionally, this case highlights an incidence of TPP in the Polynesian population, a group that is under-researched and under-represented in medical literature. We hope to shed light on the occurrence of TPP in Polynesian populations in the United States and contribute to the existing medical literature on neurological and endocrine disorders in Polynesian populations. While rare, TPP should be considered in all patients with muscle weakness or paralysis alongside hypokalemia, especially in young to middle-aged men of Asian and Polynesian descent.

## References

[1] Barahona MJ, Vinagre I, Sojo L, Cubero JM, Pérez A (2009). Thyrotoxic periodic paralysis: a case report and literature review. Clin Med Res.

[2] Patel M, Ladak K (2021). Thyrotoxic periodic paralysis: a case report and literature review. Clin Med Res.

[3] Stedwell RE, Allen KM, Binder LS (1992). Hypokalemic paralyses: a review of the etiologies, pathophysiology, presentation, and therapy. Am J Emerg Med.

[4] Salih M, van Kinschot CM, Peeters RP (2017). Thyrotoxic periodic paralysis: an unusual presentation of hyperthyroidism. Neth J Med.

[5] Hsieh CH, Kuo SW, Pei D (2004). Thyrotoxic periodic paralysis: an overview. Ann Saudi Med.

[6] Allard M, Barrallier M, Pisaroni H (2023). Thyrotoxic periodic paralysis associated with lactic metabolic acidosis: case report of an African man and review of literature. Ann Endocrinol (Paris).

[7] Lin SH (2005). Thyrotoxic periodic paralysis. Mayo Clin Proc.

[8] Elston MS, Orr-Walker BJ, Dissanayake AM, Conaglen JV (2007). Thyrotoxic, hypokalaemic periodic paralysis: Polynesians, an ethnic group at risk. Intern Med J.

[9] Carroll DG (1994). Hypokalaemic periodic paralysis in a thyrotoxic Polynesian. Postgrad Med J.

[10] Brown JD, Kangwanprasert M, Tice A, Melish J (2007). Thyrotoxic periodic paralysis in a Polynesian male following highly active antiretroviral therapy for HIV infection. Hawaii Med J.

[11] Soonthornpun S, Setasuban W, Thamprasit A (2009). Insulin resistance in subjects with a history of thyrotoxic periodic paralysis (TPP). Clin Endocrinol (Oxf).

[12] Heydorn A, Bertelsen B, Nolsöe RL, Eiken P, Kristensen PL (2023). Thyrotoxic periodic paralysis in a Caucasian man without identifiable genetic predisposition: a case report. Thyroid Res.

[13] Chang CC, Cheng CJ, Sung CC, Chiueh TS, Lee CH, Chau T, Lin SH (2013). A 10-year analysis of thyrotoxic periodic paralysis in 135 patients: focus on symptomatology and precipitants. Eur J Endocrinol.

[14] Scappaticcio L, Trimboli P, Keller F, Imperiali M, Piccardo A, Giovanella L (2020). Diagnostic testing for Graves' or non-Graves' hyperthyroidism: a comparison of two thyrotropin receptor antibody immunoassays with thyroid scintigraphy and ultrasonography. Clin Endocrinol (Oxf).

[15] Haugen BR, Alexander EK, Bible KC (2016). 2015 American Thyroid Association management guidelines for adult patients with thyroid nodules and differentiated thyroid cancer: the American Thyroid Association guidelines task force on thyroid nodules and differentiated thyroid cancer. Thyroid.

